# Patient and public involvement in cancer care and research: the Indian perspective

**DOI:** 10.3332/ecancer.2026.2084

**Published:** 2026-03-09

**Authors:** Priya Ranganathan, Manju Sengar, Aparna Mittal, Vivek Tomar, Girish Chinnaswamy, C S Pramesh

**Affiliations:** 1 Department of Anaesthesiology, Critical Care and Pain, Tata Memorial Hospital, Tata Memorial Centre, Homi Bhabha National Institute, Mumbai 400094, India; 2 National Cancer Grid, Homi Bhabha Block, Tata Memorial Hospital, Mumbai 400012, India; 3 Department of Medical Oncology, Tata Memorial Hospital, Tata Memorial Centre, Homi Bhabha National Institute, Mumbai 400094, India; 4 PatientsEngage Health Foundation, Mumbai 400013, India; 5 Rise To Survive Cancer, Gurugram 122004, India; 6 Division of Thoracic Surgery, Department of Surgical Oncology, Tata Memorial Hospital, Tata Memorial Centre, Homi Bhabha National Institute, Mumbai 400094, India

**Keywords:** patient advocacy, patient representative, public participation, community involvement

## Abstract

Biomedical research aims at improving human health through scientific studies, and is crucial for medical progress through identifying more effective ways to prevent, diagnose and treat disease. Patients (and public) are integral elements in medical research, and no research is meaningful unless it is patient (or person) centered. However, conventionally, the role of patients and the public has been restricted to only being participants in research. There is growing awareness that to be truly person-centered, research must include the patient and public voice at every stage of the research process, including priority setting, asking the right question, designing and conducting the study, interpreting and disseminating the results and ensuring that research informs policy. While this framework is reasonably well-established in some high-resource countries, it is at a very nascent stage in resource-poor settings. In this article, we examine the role of patient and public involvement in cancer care and research, with a focus on the Indian perspective.

## Background

Patient and public involvement in shaping healthcare services and research has stemmed from concerns regarding the failure of systems to acknowledge the user viewpoint and instead adopting a paternalistic approach. Though it seems logical that patients and public should have a voice in any matter that concerns their health at a policy and health system level, it has taken several decades to put systems and processes in place to address this issue, even in high-income countries (HICs); these are far less mature in low and middle income countries (LMICs) like India. Involving patients and public in healthcare decisions not only ensures user centricity but also helps in reducing the growing lack of confidence and skepticism towards research integration in healthcare systems [1].

One of the ways to bring forward the issues faced by patients is through patient advocacy. Patient advocates include individuals who have faced illness themselves, or have cared for their loved ones, non-profit organisations that work on specific disease areas and professionals who work towards empowering patients. They work towards sharing knowledge about disease, treatment options, information on what the journey with illness would be, creating a link between healthcare professional and patients, identifying funding options, ensuring dignity in care, providing emotional support and advocating for access [2]. These efforts have matured significantly in HICs and have successfully led to the involvement of patient advocacy groups in promoting equity and access in healthcare. The role of patient advocates in drug development and approval started with HIV/AIDS in the 1980s which led to the inclusion of patients on advisory committees in the Food and Drug Administration [3]. An example of how strong advocacy efforts can translate to drug approvals is reflected in the US FDA approval of eteplirsen for the treatment of Duchenne muscular dystrophy, where no effective treatment was available [4]. The accelerated approval was based on a very small study and sparked considerable debate and controversy, but the strong views from patient advocates resulted in conditional approval with the requirement of an additional larger study.[5] Another illustration of the impact of patient advocacy is the case of the price of insulin, which was slashed substantially due to their efforts [6]. These examples highlight the power of thoughtful and concerted efforts from patient advocates who have an in-depth understanding of the issues faced by patients and have knowledge of navigating the complexities of healthcare and policies.

## Patient advocacy in India

In India, patient and public involvement in health care has grown tremendously over the last decade, but has largely been restricted to advocacy for disease awareness and support groups for treatment. For example, the Organisation for Rare Diseases in India (ORDI) is a not-for-profit organisation addressing challenges faced by patients with rare diseases [7]. PatientsEngage is a moderated platform that builds health literacy among persons living with chronic conditions and their family caregivers and helps them make informed decisions about managing their condition more effectively [8]. The content is built around insights gained from speaking with thousands of people with lived experience across multiple conditions. Another patient support group in the cancer domain is V Care, a strong volunteer support group dedicated to providing cancer care, awareness and education to individuals and care givers and facilitated access to sunitinib through patient access programs for renal cancer [9]. Rise to Survive Cancer is a patient advocacy initiative to ensure access to novel therapies, helping patients to navigate and access the right treatments [10]. Similarly, ImPaCCT Foundation covers an entire spectrum to support patients with childhood cancer, including financing treatment, education of children and psychosocial support [11]. The Hematology Cancer Consortium has a group of patient advocates to address the information needs of patients suffering from hematological malignancies [12]. Examples of other organisations working for patient advocacy include the Bharath MD foundation, Scleroderma Foundation India, Metabolic Errors and Rare Diseases Organisation of India, Multiple Sclerosis Society of India, Thalassemia patient advocacy group, Hemophilia federation, Kidney Warriors Foundation and Blue Circle Diabetes [13–20].

## Patient and public involvement (PPI) in biomedical research

For research to be meaningful and impactful, it must address the priorities of those affected by the disease, and this is not possible without involving them. Therefore, a key area for PPI is biomedical research. PPI refers to the practice of engaging individuals with lived experience of illness, and members of the public, to actively participate in various stages of the research process to ensure that the research is meaningful and relevant for patients. For cancer research, these individuals could include patients with cancer, cancer survivors or caregivers of patients with cancer. PPI should engage patient representatives at every aspect of the research cycle, from the identification and prioritisation of research questions, commissioning, designing and managing research projects, dissemination of results and implementation and impact assessment. Involvement as part of the research steering committees can align the research topics to what is relevant for patients with the disease in question. The Priority III qualitative study explored public involvement in research priority setting, highlighted unique aspects, views and solutions imparted by public partners even though they felt out of their comfort zone [21]. PPI also helps to ensure that the design, methods and outcomes of research study protocols are patient-centered [22]. For example, the use of inappropriate comparators, very frequent investigations or follow-up visits or clinically irrelevant outcome measures affects compliance to the protocol, the generalisability of the study results and importantly, implementation in real-life. Clinical trials, especially those testing investigational new drugs, often have stringent and restrictive inclusion criteria. While this creates a homogeneous population and increases the internal validity of the results, it also leads to under-representation of marginalised populations, decreased external validity and slow participant accrual. Here, PPI helps to optimise eligibility criteria and identify barriers to enrollment and retention. The involvement of local patient representatives allows recognition of socio-cultural beliefs and practices that may be a deterrent to participation in research. It also helps researchers understand the burden of care (including time and financial toxicity) experienced by patients and their families who will be enrolled. This may be particularly important in global trials where the research protocol is developed in a different setting.

The inclusion of patient representatives helps ensure checks throughout the conduct of the trial, protecting participants’ interests. Patient advocates can review participant material such as study-related information, consent documents and communications, helping to simplify these for ease of understanding. They are sometimes involved in the development of meaningful patient-reported outcome measure tools. Finally, patient representation ensures that research findings are interpreted and disseminated in a non-technical, unbiased and comprehensible manner. In most HICs, research funding agencies require patient representatives to be an integral part of research projects right from inception. Increasingly, but only slowly, they are also part of defining the focus areas of research based on what matters to the groups they are representing. PPI in research results in amplification of the patient voice and democratisation of the research process, a change from the traditional hierarchical researcher–patient relationship [23]. The inclusion of PPI has been shown to result in increased accrual of research participants and may lead to improved retention and better quality of research [24, 25].

Patient representatives should have meaningful and measurable participation in research to have a real impact. To avoid biased inputs, researchers need to ensure the inclusion of multiple patient representatives from diverse sectors of society, especially marginalised sections. However, it is often difficult to identify patient advocates who have sufficient scientific and research knowledge to provide relevant inputs. Nevertheless, patient advocates in HICs are able to effectively advocate in spite of having limited scientific knowledge. Successful partnership of researchers and public requires a safe space, support and understanding of complex research methodology and terminology. This mandates training to familiarise patient and public partners in research and medical terminologies, research methods, process, ethics, good clinical practice, how to review documents and how to give inputs, keeping in mind the broader patient group with the disease. It is also essential for the researcher to view the advocate as an expert by lived experience and actively listen to their views. Occasionally, there may be a conflict between the views of the researcher and the advocate. For example, to establish scientific validity, a research study may require placebo control or collection of substantial amounts of tissue, which may not be acceptable from the patient perspective. Sometimes, research protocols may increase the burden of care among participants already struggling with multiple challenges. There have been examples of research ideas being abandoned due to a lack of consensus between researchers and patient advocates [26]. In situations where the research idea was not person-centric, this could be a good filter to screen studies. Researchers need to ensure that the inclusion of patient advocates in research achieves its goal of active participation, instead of being mere tokenism. In addition, there is a need for appropriate compensation for their time and efforts, and this needs to be dealt with care to ensure no conflicts or bias.

## Global resources for PPI

Several international organisations have set up programs to promote PPI in biomedical research. In the United Kingdom, the NIHR Centre for Engagement and Dissemination has been set up with the aim of promoting patient and community engagement in biomedical research [27]. The International Network for Public Involvement and Engagement in Health and Social Care Research is a global partnership that shares knowledge and promotes, supports and strengthens patient and public involvement in health research [28]. The network also hosts a series of Cochrane Learning live webinars on PPI [29]. The Southwest Oncology Group, which is part of the US NCI’s National Clinical Trials Network, has developed a framework for patient engagement in research, based on principles outlined by the Patient-Centered Outcomes Research Institute [30]. The Core Outcome Measures in Effectiveness Trials initiative has a separate People and Patient Participation, Involvement and Engagement working group to look at strategies for PPI in research [31]. The Strategy for Patient Oriented Research is an initiative by the Canadian Institute of Health Research that emphasises the need for the involvement of patients in all aspects of healthcare research [32]. The Guidance for Reporting Involvement of Patients and Public (GRIPP) checklist was developed to enhance the enhance the quality, transparency and consistency of reporting of involvement of patient and public activities in healthcare [33]. It has since been updated to the GRIPP-2 checklist, which has both long and short forms for reporting involvement of patient and public activities in healthcare [34]. A recent review paper provides an exhaustive overview of various resources related to PPI [24].

## PPI in biomedical research: current status in India

A systematic review of studies examining PPI in health research in LMICs identified that PPI is largely limited to research planning and involves setting up of community advisory boards to increase participant recruitment rather than actual patient representative involvement [35]. The identified studies did not explicitly define PPI or identify requirement of PPI for funding. In contrast, another review suggested that PPI in LMICs is more during the research execution phase [25]. However, patient and public involvement in medical research in India (and other LMICs) has been sporadic at best. There are several reasons for this – first, clinical research in India is largely driven by practicing clinicians, and the dual role of clinician and researcher is less understood by patients, who largely look at clinicians as those who provide care. Often, the hierarchical relationship deters patients from voicing their opinion and concerns. In addition, clinicians who are not actively involved in research do not encourage conversations about potential participation in clinical trials. Second, biomedical research is largely seen by the Indian public and patients as something done in HICs and merely implemented in LMICs; there are also negative perceptions of research as ‘experimentation’ and of participants as ‘guinea pigs’. Third, local research funding agencies in LMICs do not mandate the inclusion of patient representatives in research, unlike HICs where PPI is essential to be eligible for research funding. Fourth, the cultural diversity, literacy levels and lack of training programs for the public and patients are some of the other hurdles to deliver effective PPI in research. Finally, the biomedical research community has not done enough to disseminate the fact that research is critical for medical progress, and that patients and public should participate actively in all stages of the process.

In the recent past, organisations like the Indian Society for Clinical Research (ISCR) and the National Cancer Grid (NCG) have taken several initiatives to address these issues. The ISCR is an association of clinical research professionals from India, including academic and industry partners [36]. The ISCR runs clinical research awareness programs to coincide with International Clinical Trials Day on 20th May 20 every year, reaching out to thousands of participants. The ISCR also conducts training sessions on good clinical research practice and the current research regulations in India, where anyone likely to be involved in biomedical research can get trained. The ISCR has developed awareness videos regarding clinical research in English and other common Indian languages, which are disseminated to the public in meetings and hospital waiting rooms. In addition, the ISCR has several activities for the lay public, including quizzes, slogan competitions, marathons, poster sessions and newsletters, which are centered on the theme of clinical research.

The NCG in India is a network of more than 375 cancer centres, research institutes, patient groups, professional societies and charitable institutions across India with the mandate of establishing uniform standards of patient care for prevention, diagnosis and treatment of cancer, providing specialised training and education in oncology and facilitating collaborative basic, translational and clinical research in cancer [37–39]. Early in its evolution, the NCG identified the relative lack of patient and public involvement in biomedical research as a gap (and an opportunity) to be addressed [40]. Several initiatives have subsequently been taken by the NCG in bridging these gaps. The NCG now requires that researchers funded by them must include patient or public representatives in their research teams. This has resulted in active PPI in all recent studies funded by the NCG. The NCG organises an annual training course – the International Collaboration for Research Methods Development in Oncology (CReDO) workshops to mentor early career researchers on the methods of clinical cancer research [41]. In recent CReDO workshops, Indian and international patient representatives have been embedded as an integral part of protocol development groups, providing useful suggestions to researchers developing their research studies. This initiative also builds capacity in patient and public representation in research in India, which are otherwise a scarce resource.

An important recent initiative by the NCG is a partnership with PatientsEngage to develop a training program to empower patients and caregivers with lived experiences to effectively function as patient advocates. Through a mix of virtual and in-person sessions, this program aims to provide training on the fundamentals of oncology, basics of clinical research and essentials of patient advocacy, as well as aspects of guideline development, proposal review, communication skills, bioethics and nuances of value-based care. The virtual sessions are followed by in-person training to expand on these concepts through case studies and participation in research protocol development with oncologists and other patient advocacy mentors. The program provides mentorship by researchers, patient advocates, oncologists and research ethicists from across the world. This training has provided considerable impetus in building the much-needed resource for PPI in India.

There is a need to develop contextually relevant guidelines for PPI for India, which can guide researchers and those who are passionate about getting involved as patient or public representatives in various aspects of research. This training should include fundamentals of quality improvement, implementation research, basic science and clinical trials. These guidelines should lay down the framework for PPI at each stage, including strategies to identify suitable representatives, resources available for training and assessment, guidance on reasonable compensation for time and efforts, along with monitoring and evaluation to assess the impact of PPI on research in India. These guidelines should also define the mechanism for patient groups to work with the research community on topics that are meaningful to them.

## Conclusion

The growing burden of cancer in India is compounded by a wide variation in resources and expertise. There are efforts from the government and large organisations to improve access to standardised cancer care, driven by evidence-based guidelines and locally conducted research. To further enhance this, it becomes extremely important to bring the patient and public voice into all aspects of care delivery. Building a community of practice with this group based on a well-defined framework of training, engagement and empowerment would ensure that cancer care is meaningful and value-based for all.

## Conflicts of interest

Nil.

## Funding

Nil.
